# Seasonality, Epidemiology and Outcome of Congenital Diaphragmatic Hernia in South West of Iran

**DOI:** 10.21699/jns.v6i2.506

**Published:** 2017-04-15

**Authors:** Masoud Dehdashtian, Shiva Bashirnejad, Arash Malekian, Mohammad Reza Aramesh, Mohammad Hasan Aletayeb

**Affiliations:** Department of pediatrics (Neonatal Intensive Care Unit); Imam Khomeini hospital; Ahvaz Jundishapur University of Medical Sciences; Iran

**Keywords:** Congenital Diaphragmatic Hernia, Risk Factors, Seasonal variations.

## Abstract

**Introduction:**

The pathogenesis of congenital diaphragmatic hernia (CDH) is not clear. Risk factors including environmental factors have been implicated in the pathogenesis of few congenital anomalies. We aimed to assess the effect of season on the incidence of CDH and mortality rate in the southwest of Iran.

**Material and Methods:**

In this retrospective study, the records of 60 patients with CDH who were admitted at Neonatal Intensive Care Unit (NICU) of Imam Khomeini Hospital of Ahvaz, Iran were evaluated.

**Results:**

Assuming that all the neonates born with CDH in the region reach this hospital, overall CDH prevalence rate was 1.09 per 10 000 total births. Conceptions in spring and summer in this region had statistically significantly higher incidence of CDH. Survival rate in the series was 41.6%.

**Conclusion:**

Seasonal variation has impact on the incidence of CDH. Mortality rate in neonates with CDH is still very high.

## Introduction

Congenital Diaphragmatic Hernia (CDH) is a malformation of the diaphragm, that secondary to persistence of a defect in the diaphragm, the abdominal contents protrude into the thoracic cavity [1]. Development of the diaphragm starts during the 4^th^ week of gestation and completed by 8^th^ weeks. But in the fetuses with CDH, the diaphragm doesn't close completely during this period [2]. The prevalence of CDH is reported to be from 2.4 to 4.9 per 10000 births [3,4]. The pathogenesis and etiology of CDH is complex and remains poorly understood. Nitrofen and retinoids has been known to induce CDH in animal models [5-9]. Prenatal exposure to various maternal factors such as alcohol, smoking, peri-conceptional low intake of retinol, obesity and antimicrobial drugs have been also indicted [1,10,11]. Genetics also probably plays a role [12]. 

McKeown and Record in 1951 performed one of the earliest analysis of the seasonal pattern in the prevalence of congenital malformation and found that more cases of anencephaly were born in the autumn and winter [13]. Many perinatal risk factors for the development of congenital malformation may be seasonal such as air pollution, influenza virus, other infectious agents, dietary intake, and pesticides [14].

The survival rate for CDH in the United Kingdom was reported to be 64.6% at 1 week, 58.4% at 1 year and 57.1% at 20 years [15]. 

The aims of this study are to assess the effect of season on the incidence and epidemiology and outcome of CDH in the southwest of Iran. 

## Materials and Methods

Imam Khomeini Hospital of Ahvaz is the only referral center in the Khuzestan province of Iran having neonatal intensive care unit (NICUs) and pediatric surgical services. We did a retrospective outcome analysis of the neonates presenting to us with CDH over a 5-year period (March 2011 to February 2016). Maternal factors (age, the route of delivery, month of conception) and neonatal demographic characteristics (sex, gestational age, birth body weight) were recorded. The laterality of CDH was noted. Time of onset of respiratory distress, beginning of intubation and of mechanical ventilation, date of operation (days after birth) were also noted. Nitric oxide, high frequency oscillatory ventilation (HFVO) and Extra-corporeal Membrane Oxygenation (ECMO) were not available in our center; the neonates complicated with pulmonary hypertension were treated with hyperventilation, milrinone or magnesium sulfate.

### Statistical analysis

Descriptive analysis was performed. Data are reported as frequencies, means and standard deviation. Associations of selected variables with the outcomes were assessed with chi-squared tests, logistic regression models and proportional odds models.

## Results

Of the 548052 live births over the 5-year-period in the Khuzestan province, 60 neonates with confirmed diagnosis of CDH were admitted at NICU of Imam Khomeini Hospital of Ahvaz. With an assumption that no CDH was missed, it gives an overall CDH prevalence rate of 1 per 10000 total births. There was slightly higher incidence noted for males; M: F ratio being 7:5 (Table 1). Mean birth weight was 2944.17±75 gm, (range 1100- 4100). Majority of the CDH were left-sided with only 4 being right-sided; it was bilateral in one neonate. 

This study shows the effect of seasonal variation on the prevalence of congenital diaphragmatic hernia in Khuzestan (Tables 2, 3).

Nine (26.4%) of patients succumbed after surgery (Table 4). The patients that survived were hospitalized for 17.96± 2.13 days (range 8- 55 days). Finally, 25 (41.6%) of patients discharge alive from hospital. However, the patients were not followed after discharge from the hospital. 

## Discussion

Conceptions in spring and summer were known to be significantly associated with increased prevalence of CDH in this study. The effect of season on the prevalence of CDH has been reported earlier too [16]. CDH is known to occur more frequently after maternal influenza/fever during the first trimester [14]. Seasonal peaks (January) corresponding with influenza season was detected for CDH, but this association did not reach statistical significance [17]. However, other studies did not find any association between seasonal variation and prevalence of diaphragmatic hernia [18-20]. 

Ahvaz has the world's worst air pollution according to a survey by the World Health Organization in 2011 [21]. This situation is exaggerated by drought and reduced rainfall and soil moisture during spring and specially summer. One of the major origins of this air pollution is Iraq. It has been suggested that war-created pollution is a major cause for increasing the prevalence of birth defects in Iraq [22]. 

In this study, the prevalence of CDH was 1.09 per 10000 live born infants; this is far lower than other studies. In a study including only live birth neonates prevalence of CDH were reported 2.38 per 10000 [23]. Another study that includes live births, stillbirths, miscarriages, termination of pregnancy. The prevalence of CDH in another study that includes live births, stillbirths, miscarriages, termination of pregnancy was reported 4.90 per 10000 [4]. The probable reason for the low prevalence of CDH in our study could be that we don’t receive all the neonates; the sicker ones die before they are referred to our facility. The sex ratios calculated in this study are comparable to the highest ratios previously reported in the literature [24, 25]. 

This study showed that survival significantly affected by delivery route. There are several reports that elective cesarean section is associated with persistent pulmonary hypertension [26, 27]. No statistically significant associations were found in any maternal age categories, which is compatible with the previous study [3]. Survival in term babies was significantly higher than in preterm babies; this fact is universally acknowledged [28]. Early presentation of respiratory distress significantly affected the survival of patients that may be a reflection of the severity of lung hypoplasia or pulmonary hypertension. The side of herniation did not affect the survival of our patients; this is in contrast with an earlier study by Grizelj et al [29]. Survival rate of 41.6% in our series is lesser than survival rate among neonate with that reported from Croatia, France, England, America -47.9%, 47%, 69% and 81% at discharge respectively [28-31]. Higher mortality rate in our center probably results from inaccessibility to nitric oxide, HFO, and ECMO.

## Conclusion

The incidence of CDH in the southwest of Iran is 1.09 per 10 000 total births, which is so less than reported prevalence of other countries. Also conceptions in spring and summer were significantly associated with increased prevalence of congenital diaphragmatic hernia. Mortality of newborns with congenital diaphragmatic hernia in the southwest of Iran is higher than reported studies of other countries with modern technology.

## Footnotes

**Source of Support:** None

**Conflict of Interest:** None

## Figures and Tables

**Table 1:** Characteristics of neonates with CDH.

Characteristics	Number (%)
Sex	
Male	35 (58.3)
Female	25 (41.7)
	
Gestational age(week)	
≥37	47 (78.3)
≥34 and <37	7 (11.7)
<34	6 (10)
	
Birth Weight (g)	2944.17 75
Range	1100- 4100
	
Side of herniation	
Left	55 (91.7)
Right	4 (6.7)
bilateral	1 (1.7)
	
Onset of respiratory distress(h)	
<6	44 (73.3)
>6 to <24	3 (5)
≥24	13 (21.7)
	
Initiation of mechanical ventilation- after birth (h)	
at birth	13 (21.6)
<6	43 (71.6)
6- 24	1 (1.6)
>24	3 (5)

**Table 2:** The effect of season and month of last menstrual period on the prevalence of CDH.

Season and month	CDH cases	*P*
Spring	21	0.009
April	6	
May	11	
June	4	
	
Summer	22	
July	4	
August	9	
September	9	
	
Fall	7	
October	1	
November	4	
December	2	
	
Winter	10	
January	4	
February	2	
march	4	

**Table 3:** odd ratio for prevalence of congenital diaphragmatic hernia in different seasons

	OR	95%CI	*P*
Spring-Summer	1.016	(0.557,1.855)	*0.95*
Spring-Fall	3.236	(1.374,7.634)	*0.00*
Spring-Winter	2.051	(0.963,4.368)	*0.06*
Summer-Fall	3.188	(1.358,7.481)	*0.00*
Summer-Winter	2.017	(0.952,4.272)	*0.06*
Fall-Winter	0.633	(0.241,1.666)	*0.35*

**Table 4:** Patients characteristic effect on the neonatal outcome

Variable	Alive	DBS	DAS	*P*
Delivery route				
Unknown	0	1	1	*0.008*
Cesarean	12	21	2	
Vaginal	12	4	6	
				
Maternal age (yr)				
<20	4	6	2	*0.18*
20- 30	13	9	6	
>30	8	7	5	
				
Gestational age(wk)				
≥;37	23	15	9	*P=0.01*
34-36	2	5	0	
<34	0	6	0	
				
Onset of R.D (hr)				
<6	9	26	9	*0.00*
6- 24	3	0	0	
>24	12	0	0	
				
Side of herniation				
Left	23	24	9	*0.49*
Right	3	1	0	
Bilateral	0	1	0	

## References

[1] Leeuwen L, Fitzgerald DA (2014). Congenital diaphragmatic hernia. J Paediatr Child Health.

[2] Langham M R, Kays D W, Ledbetter D J  (1996). Congenital diaphragmatic hernia epidemiology and outcome. Clin Perinatol.

[3] McGivern MR, Best KE, Rankin J, Wellesley D, Greenlees R, Addor MC  (2015). Epidemiology of congenital diaphragmatic hernia in Europe: a register-basedstudy. Arch Dis Child Fetal Neonatal Ed.

[4] Tennant PW, Pearce MS, Bythell M et a (2010). 20-year survival of children born with congenital anomalies: a population-based study. Lancet.

[5] Allan DW, Greer JJ (1997). Pathogenesis of nitrofen-induced congenital diaphragmatic hernia in fetal rats. J Appl Physiol.

[6] Andersen DH (1941). Incidence of congenital diaphragmatic hernia in the young of rats bred on a diet deficient in vitamin A. Am J Dis Child.

[7] Wilson JG, Roth CB, Warkany J (1953). An analysis of the syndrome of malformations induced by maternal vitamin A deficiency. Effects of restoration of vitamin A at various times during gestation. Am J Anat.

[8] Greer JJ, Babiuk RP, Thebaud B (2003). Etiology of congenital diaphragmatic hernia: the retinoid hypothesis. Pediatr Res.

[9] Caspers KM, Oltean C, Romitti PA, Sun L, Pober BR, Rasmussen SA  (2010). Maternal periconceptional exposure to cigarette smoking and alcohol consumption and congenital diaphragmatic hernia. Birth Defects Res A Clin Mol Teratol.

[10] Beurskens LW, Tibboel D, Steegers-Theunissen RP (2009). Role of nutrition, lifestyle factors, and genes in the pathogenesis of congenital diaphragmatic hernia: human and animal studies. Nutr Rev.

[11] Crider KS, Cleves MA, Reefhuis J, Berry RJ, Hobbs CA, Hu DJ (2009). Antibacterial medication use during pregnancy and risk of birth defects: National Birth Defects Prevention Study. Arch. Pediatr Adolesc Med.

[12] McKeown T, Record RG (1951). Seasonal incidence of congenital malformations of the central nervous system. Lancet.

[13] Luteijn JM, Dolk H, Addor MC, Arriola L, Barisic I, Bianchi F  (2014). Seasonality of congenital anomalies in Europe. Birth Defects Res A Clin Mol Teratol.

[14] Cilley RE, Zgleszewski SE, Krummel TM, Chinoy MR (1997). Nitrofen dose-dependent gestational day-specific murine lung hypoplasia and left-sided diaphragmatic hernia. Am. J. Physiol.

[15] Caton AR (2012). Exploring the seasonality of birth defects in the New York State Congenital Malformations Registry. Birth Defects Res A Clin Mol Teratol.

[16] Czeizel AE, Puhó EH, Acs N, Bánhidy F (2008). Use of specified critical periods of different congenital abnormalities instead of the first trimester concept. Birth Defects Res A Clin Mol Teratol.

[17] Bound JP1, Harvey PW, Francis BJ (1989). Seasonal prevalence of major congenital malformations in the Fylde of Lancashire 1957-1981. J Epidemiol Community Health.

[18] Castilla EE, Orioli IM, Lugarinho R, Dutra GP, Lopez-Camelo JS, Campana HE  (1990). Monthly and seasonal variations in the frequency of congenital anomalies. Int J Epidemiol.

[19] Torfs CP, Curry CJ, Bateson TF, Honoré LH (1992). A population-based study of congenital diaphragmatic hernia. Teratology.

[20] Walsh Bryan, (27 September 2011) (2016). "The 10 Most Air-Polluted Cities in the World" Time. Available from http://science.time.com/2011/09/27/the-10-most-air-polluted-cities-in-the-world/ Accessed on 1-12-2016.

[21] Kaiser JR, Rosenfeld CR (1999). A population-based study of congenital diaphragmatic hernia: impact of associated anomalies and preoperative blood gases on survival. J Pediatr Surg.

[22] Savabieasfahani M, Ali SS, Bacho R, Savabi O, Alsabbak M (2016). Prenatal metal exposure in the Middle East: imprint of war in deciduous teeth of children. Environ Monit Assess.

[23] Stege G, Fenton A, Jaffray B (2003). Nihilism in the 1990s: the true mortality of congenital diaphragmatic hernia. Pediatrics.

[24] Dott MM, Wong L-YC, Rasmussen SA (2003). Population-based study of congenital diaphragmatic hernia: risk factors and survival in Metropolitan Atlanta, 1968-1999. Birth Defects Res A Clin Mol Teratol.

[25] Yang W, Carmichael SL, Harris JA  (2006). Epidemiologic characteristics of congenital diaphragmatic hernia among 25 million California births, 1989-1997. Birth Defects Res A Clin Mol Teratol.

[26] Heritage CK, Cunningham MD (1985). Association of elective repeat cesarean delivery and persistent pulmonary hypertension of the newborn. Am J Obstet Gynecol.

[27] Keszler M, Carbone MT, Cox C, Schumacher RE (1992). Severe respiratory failure after elective repeat cesarean delivery: a potentially preventable condition leading toextracorporeal membrane oxygenation. Pediatrics.

[28] Wright JC, Budd JL, Field DJ, Draper ES (2011). Epidemiology and outcome of congenital diaphragmatic hernia: a 9-year experience. Paediatr Perinat Epidemiol.

[29] Grizelj R, Bojanić K, Vuković J, Novak M, Rodin U, Ćorić T  (2016). Epidemiology and Outcomes of Congenital Diaphragmatic Hernia in Croatia: A Population-Based Study. Paediatr Perinat Epidemiol.

[30] Gallot D, Boda C, Ughetto S, Perthus I, Robert-Gnansia E, Francannet C  (2007). Prenatal detection and outcome of congenital diaphragmatic hernia: a French registry-based study. Ultrasound Obstet Gynecol.

[31] Zalla JM, Stoddard GJ, Yoder BA (2015). Improved mortality rate for congenital diaphragmatic hernia in the modern era of management: 15 year experience in a single institution. J Pediatr Surg.

